# Ethyl Pyruvate Induces Tolerogenic Dendritic Cells

**DOI:** 10.3389/fimmu.2019.00157

**Published:** 2019-02-07

**Authors:** Neda Djedovic, María José Mansilla, Bojan Jevtić, Juan Navarro-Barriuso, Tamara Saksida, Eva M. Martínez-Cáceres, Ðorđe Miljković

**Affiliations:** ^1^Department of Immunology, Institute for Biological Research “Siniša Stanković” University of Belgrade, Belgrade, Serbia; ^2^Immunology Division, Germans Trias i Pujol University Hospital and Research Institute, Badalona, Spain; ^3^Department of Cellular Biology, Physiology, and Immunology, Universitat Autònoma de Barcelona, Cerdanyola del Vallès, Spain

**Keywords:** dendritic cells, ethyl pyruvate, autoimmunity, tolerogenicity, immune-regulation

## Abstract

Dendritic cells (DC) are professional antigen presenting cells that have a key role in shaping the immune response. Tolerogenic DC (tolDC) have immuno-regulatory properties and they are a promising prospective therapy for multiple sclerosis and other autoimmune diseases. Ethyl pyruvate (EP) is a redox analog of dimethyl fumarate (Tecfidera), a drug for multiple sclerosis treatment. We have recently shown that EP ameliorates experimental autoimmune encephalomyelitis, a multiple sclerosis murine model. Here, we expanded our study to its tolerogenic effects on DC. Phenotypic analysis has shown that DC obtained from mice or humans reduce expression of molecules required for T cell activation such as CD86, CD83, and HLA-DR under the influence of EP, while CD11c expression and viability of DC are not affected. Furthermore, EP-treated DC restrain proliferation and modulate cytokine production of allogeneic lymphocytes. These results demonstrate that EP has the ability to direct DC toward tolDC.

## Introduction

Tolerogenic dendritic cells (tolDC) are promising candidates for the cell-based immunotherapy of autoimmune disorders, including multiple sclerosis (1). They are effective in restraining antigen-specific and allogeneic T cell responses *in vitro* and their beneficial effects have been demonstrated in the treatment of animal models of various autoimmune disorders (2). Moreover, their administration to humans has been shown safe and efficient by increasing the proportion of regulatory T cells in circulation (1). Vitamin D3 and dexamethasone are commonly used for induction of tolDC (1, 2), while a number of agents has been shown effective for the induction of tolerogenic properties over dendritic cells (DC). Ethyl pyruvate (EP) is a redox active compound that has been shown potent as an anti-inflammatory agent (3). It is a safe and simple chemical that has already been tested in humans (4). Importantly, it is a redox analog of dimethyl fumarate (Tecfidera), a drug that is approved for multiple sclerosis treatment (5). Our group has recently reported that EP ameliorates experimental autoimmune encephalomyelitis (EAE), an animal model of multiple sclerosis (6). The major pathogenic T helper (Th) cells in the central nervous system autoimmunity are interferon (IFN)-γ-producing Th1 cells and interleukin (IL)-17-producing Th17 cells (7). The beneficial effects of EP in EAE were paralleled with down-regulation of Th1/Th17 activity (6). Moreover, release/production of IL-6, tumor necrosis factor (TNF) and reactive nitrogen and oxygen species by macrophages were also inhibited by EP. Noteworthy, IL-6 is known to potentiate the resistance of effector T cells to regulatory T cells in multiple sclerosis (8), while TNF actively contributes to demyelination and axonal degeneration in neuroinflammation (9). Correspondingly, reactive oxygen and nitrogen species participate in the loss of oligodendrocytes, blood-brain barrier dysfunction, T cell infiltration, and neurodegeneration (10). Effects of EP were also observed within the CNS, where reactivity of microglia and astrocytes was reduced (11).

We were also able to demonstrate that EP down-regulated the expression of antigen presenting molecules on macrophages (6) which led us to the investigation of the effects of EP on DC as the major professional antigen-presenting cells. Here, we present that EP exerts potent tolerogenic effect on murine and human DC. It down-regulates the expression of antigen-presenting molecules on DC, restricts the production of pro-inflammatory cytokines in DC and diminishes their T cell-activating function.

## Materials and Methods

### Monocyte-Derived Human DC

Buffy coats, provided by the *Banc de Sang i Teixits* (Barcelona, Spain), were obtained from randomized healthy blood donors, following the institutional Standard Operating Procedures for blood donation and processing. Peripheral blood was obtained from untreated relapsing-remitting multiple sclerosis patients. The Ethical Committee of Germans Trias i Pujol Hospital approved the study, and all subjects gave their informed consent according to the Declaration of Helsinki (BMJ 1991; 302: 1994). Peripheral Blood Mononuclear Cells (PBMC) were isolated by Ficoll-Paque (Lymphoprep, Axis Shield, Oslo, Norway) density gradient centrifugation at 400 × g for 30 min. Recovered cells were washed twice in PBS and counted using Perfect Count microspheres (Cytognos SL, Salamanca, Spain) following the manufacturer's instructions. Establishing Monocyte-derived DCs, PBMCs were first depleted of CD3^+^ T cells using the RosetteSep™ Human CD3 Depletion Cocktail (StemCell Technologies, Seattle, WA, United States). Afterwards, monocytes were obtained by positive selection using the EasySep® Human CD14 Positive Selection Kit (StemCell Technologies). For all samples, the purity and viability of the monocyte populations were >95 and 90%, respectively, as assessed by the expression of specific markers and Annexin V and 7-Amino-actinomycin D (7AAD) labeling (BD Biosciences). Monocytes were cultured at 1 × 10^6^/ml for 6 days in X-VIVO 15 culture medium (BioWhittaker®, Lonza, Belgium) supplemented with 2% (vol/vol) heat inactivated AB human serum (BioWhittaker®, Lonza, Belgium), 2 mM L-glutamine (Sigma-Aldrich Company LTD, Saint Louis, MO, United States), 100 U/mL penicillin (Cepa S.L, Madrid, Spain), and 100 μg/mL streptomycin (Laboratorios Normon S.A, Madrid, Spain) in the presence of granulocyte-macrophage colony-stimulating factor (GM-CSF: 1,000 U/ml; Peprotech, Freiburg, Germany) and interleukin 4 (IL-4: 1,000 U/ml; Peprotech). Cells were replenished on day 4 with fresh medium and cytokines. To induce mature DC (mDC), DC were treated with a cocktail of TNF (1,000 U/ mL), IL-1β (10 ng/mL) (both from Peprotech); and PGE_2_ (1 μM) (Pfizer, New York, NY, United States) on day 4. Treatment with Vitamin D3 (vitDC, 1 nM, Calcijex, Kern Pharma, Terrassa, Spain) was performed on days 0 and 4, while treatment with EP (EPDC) was performed on days 2 and 4. These cells were also stimulated as mature DCs at day 4 with the cytokine cocktail. Immature DC (iDC) were not treated with the maturation cocktail on day 4. On day 6, DC were harvested and washed extensively twice, before phenotype was determined and functional assays were performed.

### Bone Marrow-Derived Murine DC

Murine DC were obtained from progenitor bone marrow cells that were flushed from femur of NOD and C57BL/6 mice (experiments were approved by the local ethics committee of the Institute for Biological Research “Sinisa Stankovic,” in accordance with Directive 2010/63/EU, N° 02-09/16). These cells were cultured in RPMI 1640 (Biological Industries, Kibbutz Beit-Haemek, Israel) supplemented with 10% FCS (PAA Laboratories), 2 mM glutamine and 1 mM sodium pyruvate (both from Sigma-Aldrich) (1 × 10^6^/mL/well in 24-well plate). Bone marrow derived dendritic cells (BMDC) were cultivated for 8 days in the presence of 20 ng/mL of GM-CSF (Peprotech or Novus, Littleton, CO), with 100 ng/mL lipopolysaccharide (LPS, Sigma-Aldrich) added for the last 24 h of cultivation for maturation. Treatment with EP was performed on days 3 and 6 (diffEP) or simultaneously with LPS (matEP). Number of viable cells was determined by trypan blue exclusion test on a LUNA-II™ Automated Cell Counter from Logos Biosystems (Gyeonggi-do, South Korea).

### *In vivo* DC Application

Experiments were approved by the local ethics committee (Institute for Biological Research “Sinisa Stankovic,” in accordance with Directive 2010/63/EU, N° 03-01/17). Murine DC were prepared as described above. mDC or EPDC were injected subcutaneously into the hind paw of female 2–3 months old C57BL/6 mice (1 × 10^6^/80 μl/mouse). Complete Freund's adjuvant (CFA) was made from incomplete Freund's adjuvant (Difco, Detroit, MI) supplemented with *M. tuberculosis* (to 5 mg/ml, Difco). Each mouse was injected subcutaneously in the hind paw with 50 μl of emulsion made from CFA mixed with equal volume of phosphate buffer saline on the following day. Popliteal lymph nodes were isolated from the mice 3 days later and subjected to collagenase V (Sigma-Aldrich) digestion. Lymph nodes were gently minced with scissors and incubated with 1 mg/mL of Collagenase V solution in RPMI 1640 at 37°C with gentle shaking for 20 min. Subsequently, the digestion was stopped with FCS and the cells were pelleted by centrifugation. Number of viable cells was determined by trypan blue exclusion test on a LUNA-II™ Automated Cell Counter. For cell tracking, DC were stained with CFSE (2 μM, Invitrogen, Carlsbad, CA, United States) prior to injection. Determination of CFSE^+^ cells among popliteal lymph node cells was performed on a CyFlow Space flow cytometer (Partec, Munster, Germany).

### CD4^+^ T Cell Isolation

CD4^+^ T cells were purified from cervical lymph nodes obtained from BALB/c mice. For purification of CD4^+^ T cells from the lymph nodes, a biotin-conjugated antibody specific for CD4 (Invitrogen) and IMagSAv Particles Plus (BD Biosciences, San Diego, CA) were used in accordance with the manufacturers' instructions. CD4^+^ T cells were stained with CFSE (2 μM) prior to cultivation with DC.

### Allostimulatory Assays

Human DC were co-cultured with allogeneic human PBMC (10^5^ cells/ well) in 96-well round-bottom plates. PBMC were co-cultured for 4 days (96 h) with MDDCs at a 1:20 ratio (DC: PBMC). Cell proliferation was determined by incorporation of 1 μCi [^3^H]-thymidine (PerkinElmer, Waltham, MA, United States) for 18 h on each well. Murine DC obtained from C57BL/6 mice were co-cultured with CFSE-labeled CD4^+^ T cells obtained from BALB/c mice (10^5^ cells/well) in 96-well round-bottom plates. CD4^+^ T cells were co-cultured for 5 days (120 h) with MDDCs at a 1:20 ratio (DC: CD4^+^ T cells). Cell proliferation was determined by the sequential loss of CFSE fluorescence, as detected by flow cytometry.

### Immunostaining and Flow Cytometry

Human DC were washed, re-suspended in 50 μl of PBS and incubated with mAbs for 20 min protected from light at room temperature (RT). After washing, acquisition was performed on a FACSCanto II flow cytometer using FACSDiva software (BD Biosciences, CA, United States). Subsequent analyses were done using FlowJo software (Tree Star, Inc., OR, United States). Samples were gated using forward (FSC) and side (SSC) scatter to exclude dead cells and debris. The following human mAbs were used: FITC-labeled mAbs: CD86 (BD Biosciences); PE-labeled mAbs: CD14 (ImmunoTools GmbH, Germany), CD40 (BD Biosciences); PE-Cyanine dye 7-labeled mAb: CD14 and CD11c (BD Biosciences); Allophycocyanin (APC)-labeled mAbs: CD83, APC-H7-labeled mAb: HLA-DR (BD Biosciences).

Murine cells were washed, re-suspended in 100 μL of PBS supplemented with 2% of mouse serum and incubated with mAbs for 30 min at 4°C. After washing, acquisition was performed with a CyFlow Space flow cytometer. Samples were gated using forward (FSC) and side (SSC) scatter to exclude dead cells and debris, as well as using FSC and FSC-W to exclude cell doublets. The following murine mAbs were used: PE-Cyanine5 labeled CD11c and CD86 (eBioscience), FITC-labeled CD40 (eBioscience).

Prior to intracellular cytokine staining, cells were stimulated with eBioscience™ Cell Stimulation Cocktail (plus protein transport inhibitors), containing phorbol myristate acetate, ionomycin and brefeldin A and monensin for 4 h, stained with anti-CD4 PerCP-Cy5.5 antibody (eBioscience), fixed and permeabilized with eBioscience™ Intracellular Fixation and Permeabilization Buffer Set and then stained for the intracellular cytokines with the following antibodies: anti-mouse antibodies against IL-17 or IFN-γ or IL-10 coupled with FITC or PE (all from eBioscience). After washing, acquisition was performed with a CyFlow Space flow cytometer. Samples were gated using forward (FSC) and side (SSC) scatter to exclude dead cells and debris, as well as using FSC and FSC-W to exclude cell doublets. Isotype-matched controls were included in all experiments (eBioscience).

### ELISA

Cell culture supernatants were obtained and centrifuged to spin down the cells. Cell-free supernatants were used in sandwich ELISAs as instructed by the producers of the antibody pairs used. Samples were analyzed in duplicates for murine TNF, murine IL-6 (R&D Systems), murine IL-1β, murine IL-12 (eBiosciences), murine IL-17 (eBioscience), human IFN-γ and human IL-10 (R&D Systems). Lower limit of detection was 30 pg/ml, whereas upper limit of detection was 10 ng/ml for all of the ELISA tests performed. Samples that showed values over the upper limit of detection were adequately diluted for the measurement. The results were calculated using standard curves made on the basis of known concentrations of the appropriate recombinant cytokines.

### Statistics

One-way ANOVA followed by Tukey's multiple comparison test or Student's *t*-test (two-tailed) were used as appropriate for statistical analysis. A *p* < 0.05 was considered statistically significant.

## Results

### EP Exerts Tolerogenic Effects on Mouse BMDC

**Figure 1:**
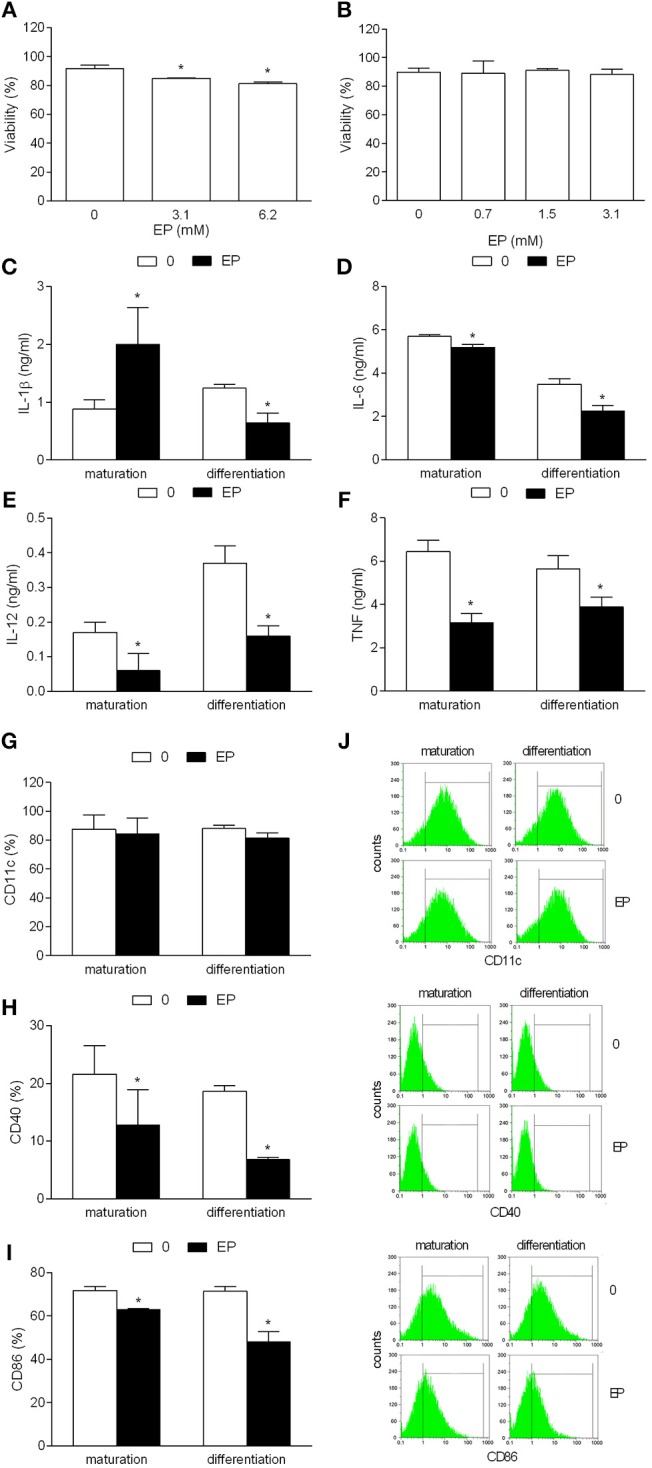
Effects of EP on NOD mice DC. BMDC were obtained from NOD mice. They were treated with EP for 24 h simultaneously with LPS (**A** and “maturation”) or at days 3 and 6 of the propagation (**B** and “differentiation”). 3.1 mM EP was used in **(C–I)**. Viability **(A,B)** was determined by trypan blue exclusion assay. Cytokines were determined in cell culture supernatants by ELISA **(C–F)**. Proportion of cells expressing the listed antigens was determined by cytofluorimetry **(G–J)**. Data are presented as mean + SD from at least three independent experiments. Data in **(J)** are representative plots. ^*^p < 0.05, EP vs. 0, one way ANOVA followed by Tukey's multiple comparisons test **(A,B)**, two-tailed *t*-test **(C–I)**.

In order to determine if EP has tolerizing effects on NOD mice BMDC in the course of their maturation, the agent was applied during the last 24 h of their cultivation, simultaneously with the maturing agent, i.e., LPS. Alternatively, to assess EP effect on differentiation/propagation of DC from their bone marrow precursors, the agent was applied to BMDC cultures on days 3 and 6 of their cultivation. EP applied during the maturation had minimal effects on BMDC viability (Figure 1A), while no effects on the cell viability were observed with the alternative application of the agent (Figure 1B). While EP applied during the differentiation reduced IL-1β production in BMDC, EP applied during maturation had the opposite effect (Figure 1C). Both ways of EP application led to the reduction of generation of IL-6, IL-12, and TNF in BMDC (Figures 1D–F). Also, CD11c expression was not affected, while both CD86 and CD40 expression were decreased in EP-treated BMDC, irrespectively of the way of its application (Figures 1G–J).

### EP-Treated Mouse BMDC Are Inefficient Allogeneic Stimulators *in vitro*

**Figure 2:**
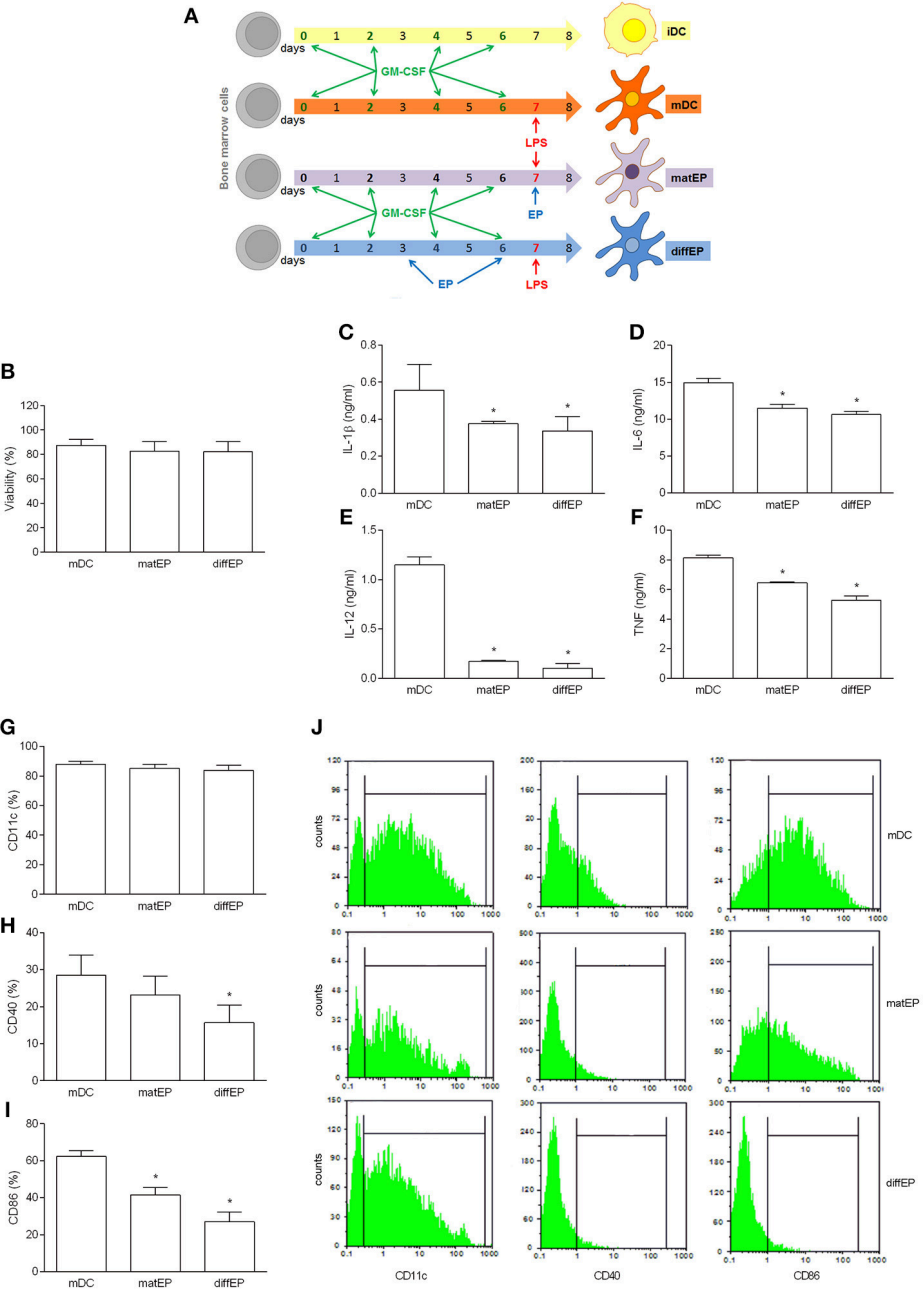
Effects of EP on C57BL/6 mice DC. BMDC were obtained from C57BL/6 mice. They were treated with EP (3.1 mM) for 24 h simultaneously with LPS (“matEP”) or at days 3 and 6 of the propagation (“difEP”) or they were untreated with EP (mDC), as schematized **(A)**. Viability **(B)** was determined by trypan blue exclusion assay. Cytokines were determined in cell culture supernatants by ELISA **(C–F)**. Proportion of cells expressing the listed antigens was determined by cytofluorimetry **(G–J)**. Data are presented as mean + SD from at least three independent experiments. Data in **(J)** are representative plots. ^*^p < 0.05, EP vs. mDC, one way ANOVA followed by Tukey's multiple comparisons test.

**Figure 3:**
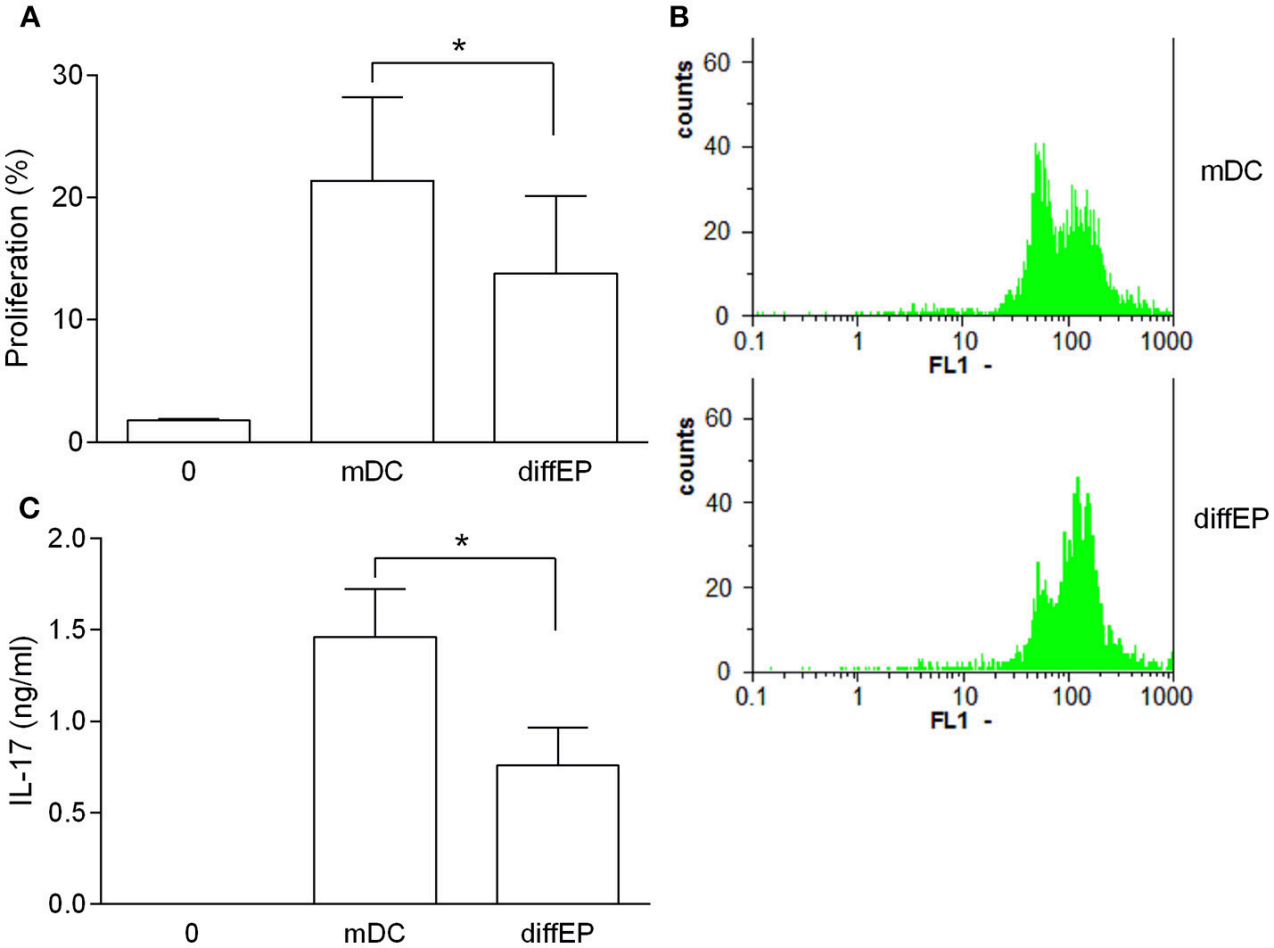
Effects of murine EP-treated DC on allogeneic reactivity. BMDC were obtained from C57BL/6 mice. Their maturation was induced by LPS in the absence (mDC) or presence of 3.1 mM EP (diffEP). They were co-cultured with CD4^+^ T cells purified from BALB/C mice lymph nodes. Cell proliferation was determined by CFSE assay **(A,B)**. IL-17 levels were determined in cell culture supernatants by ELISA **(C)**. Data are presented as mean + SD from at least three independent experiments. Data in **(B)** are representative plots. ^*^p < 0.05, diffEP vs. mDC, one way ANOVA followed by Tukey's multiple comparisons test.

To test if the tolerogenic effects of EP can also be observed in C57BL/6 BMDC, the same application protocols were used as with NOD mice BMDC. EP did not affect cell viability and CD11c expression, while it reduced generation of the examined pro-inflammatory cytokines and expression of CD40 and CD86, irrespectively of the way of its application (Figures 2A–J). To examine if these effects of EP affected BMDC ability to activate T cells, EP-treated BMDC were co-cultured with allogeneic CD4^+^ T cells from BALB/c mice. Proliferation of CD4^+^ T cells (Figures 3A,B) and their capacity to produce IL-17 (Figure 3C) were reduced in co-cultures with EP-treated BMDC in comparison to those with the untreated BMDC.

### EP Exerts Tolerogenic Effects on Human MDDC

**Figure 4:**
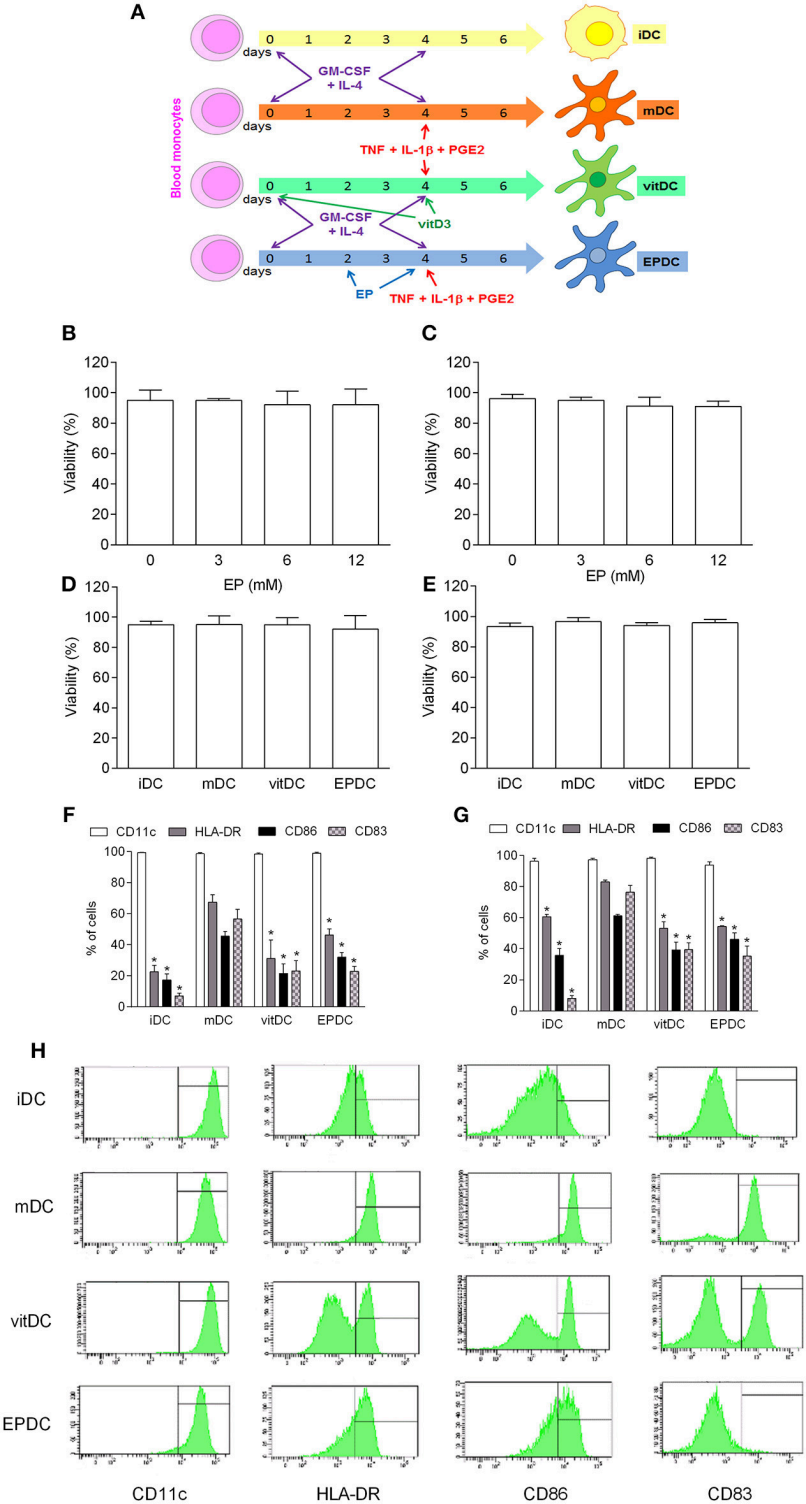
Effects of EP on human DC. MDDC were propagated from peripheral blood monocytes and matured in the presence of TNF+IL-1β+PGE_2_ (mDC, vitDC, tEPDC) or were left immature without the treatment (iDC) as depicted **(A)**. MDDC were obtained from healthy subjects **(B,D,F,H)** or from individuals affected by multiple sclerosis **(C,E,G)**. EP was applied in various concentrations **(B,C)** or in concentration of 6.2 mM (**D–H**, EPDC). Vitamin D3 (vitDC) was applied in concentration of 1 nM. Viability **(B–E)** was determined by 7AAD test. Proportion of cells expressing the listed antigens was determined by cytofluorimetry **(F–H)**. Data are presented as mean + SD from at least five individuals. Data in H are representative plots. ^*^p < 0.05 vs. mDC, two-tailed *t*-test.

EP was added to MDDC cultures in the process of their differentiation/propagation (EPDC) (Figure 4A). No effect of EP on cell viability was observed on human MDDC that were obtained from healthy subjects (Figure 4B) or individuals suffering from multiple sclerosis (Figure 4C). EP-treated DC were compared to vitamin D-treated DC (vitDC), non-treated mature DC (mDC) and immature DC (iDC) in the following experiments. There was no difference in the cell viability among the examined DC populations (Figures 4D,E). Also, no difference was observed in the level of CD11c expression (Figures 4F–H). However, both vitDC and EPDC reduced expression of HLA-DR, CD86, and CD83 in comparison to mDC, both in healthy and multiple sclerosis individuals (Figures 4F–H).

### EP-Treated Human MDDC Are Inefficient Allogeneic Stimulators *in vitro*

**Figure 5:**
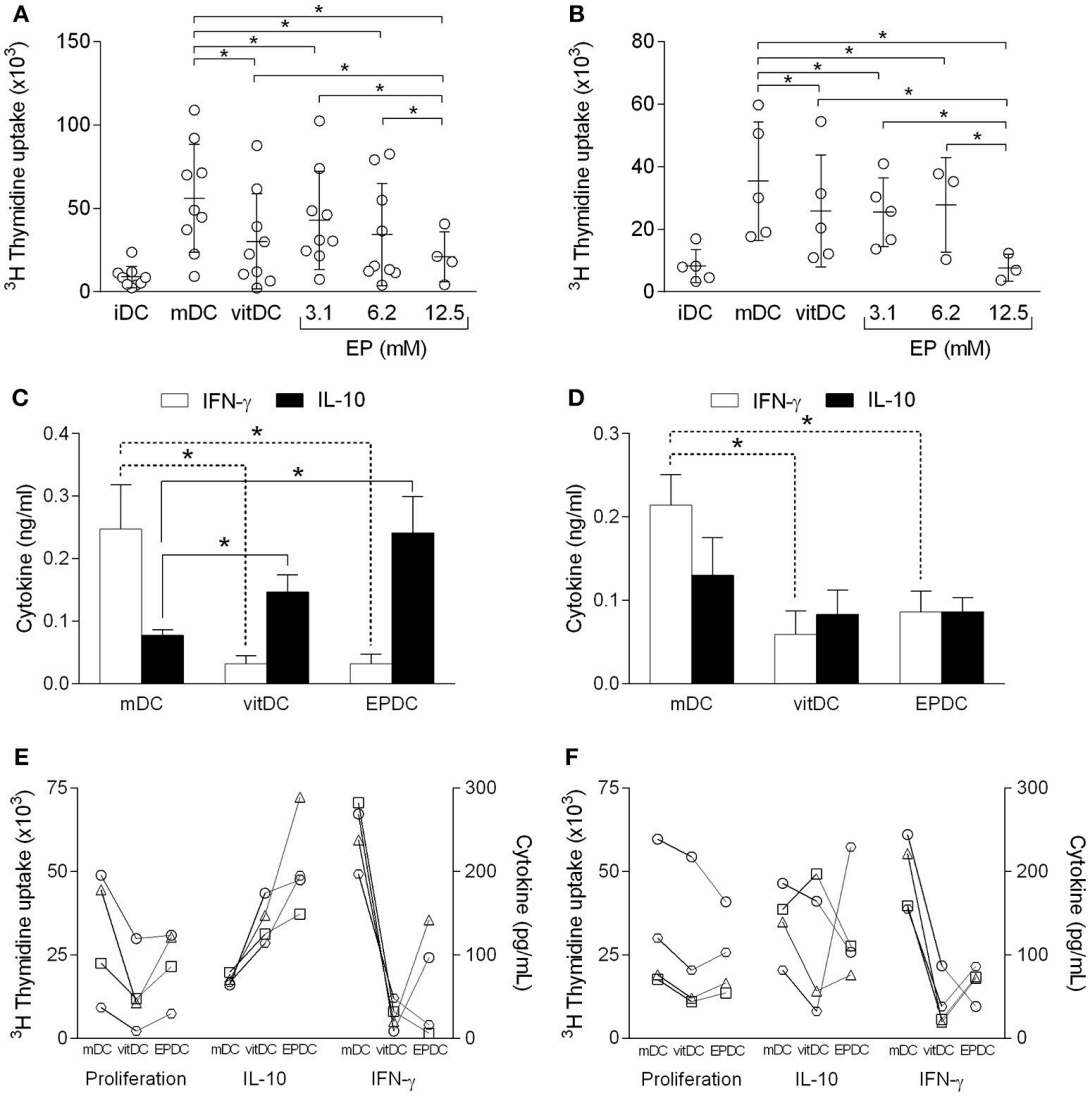
Effects of human EP-treated DC on allogeneic reactivity. MDDC were obtained from healthy subjects **(A,C,E)** or from individuals affected by multiple sclerosis **(B,D,F)**. The cells matured in the presence of TNF+IL-1β+PGE_2_ (mDC, vitDC, EPDC) or were left immature without the treatment (iDC). EP was applied in various concentrations **(A,B)** or in concentration of 3.1 mM **(C–F)**. Vitamin D3 (vitDC) was applied in concentration of 1 nM. MDDC were co-cultured with allogeneic PBMC. Cell proliferation was determined by ^3^H incorporation test **(A,B,E,F)**. Cytokines were determined in cell culture supernatants by ELISA **(C–F)**. Data in **(A,B)** are presented as mean ± SD from a number of individuals depicted as the open circles. Data in **(C,D)** are presented as mean + SD from four individuals. Data from four individuals are presented in **(E,F)**, where the same person has a unique symbol (circle, square, triangle, or trapezoid). ^*^p < 0.05, vs. mDC in **(C,D)**, one way ANOVA followed by Tukey's multiple comparisons test.

Tolerogenic properties of EPDC were examined in allogeneic co-culture with PBMC. EPDC inhibited allogeneic cell proliferation in comparison to mDC to the similar extent as vitDC (Figures 5A,B). Effects of EPDC obtained with the highest EP dose applied (12.5 mM) were superior to those of vitDC (Figures 5A,B). There was no difference in the inhibitory activity of EPDC or vitDC obtained from healthy subjects or individuals affected with multiple sclerosis. Levels of IFN-γ and IL-10 in supernatants of the co-cultures were also determined. IFN-γ levels were reduced in the samples obtained from co-cultures performed with EPDC or vitDC in comparison to mDC (Figures 5C,D), irrespectively if MDDC were obtained from healthy individuals or the patients. However, IL-10 levels were higher with EPDC or vitDC in comparison to mDC, only if MDDC were obtained from healthy subjects (Figures 5C,D). Allogeneic cell proliferation and the cytokine levels determined with DC obtained from individual persons are presented, as well (Figures 5E,F) in order to enable discrimination of responses from different donors.

### EP-Treated Mouse BMDC Show Immunomodulatory Function *in vivo*

**Figure 6:**
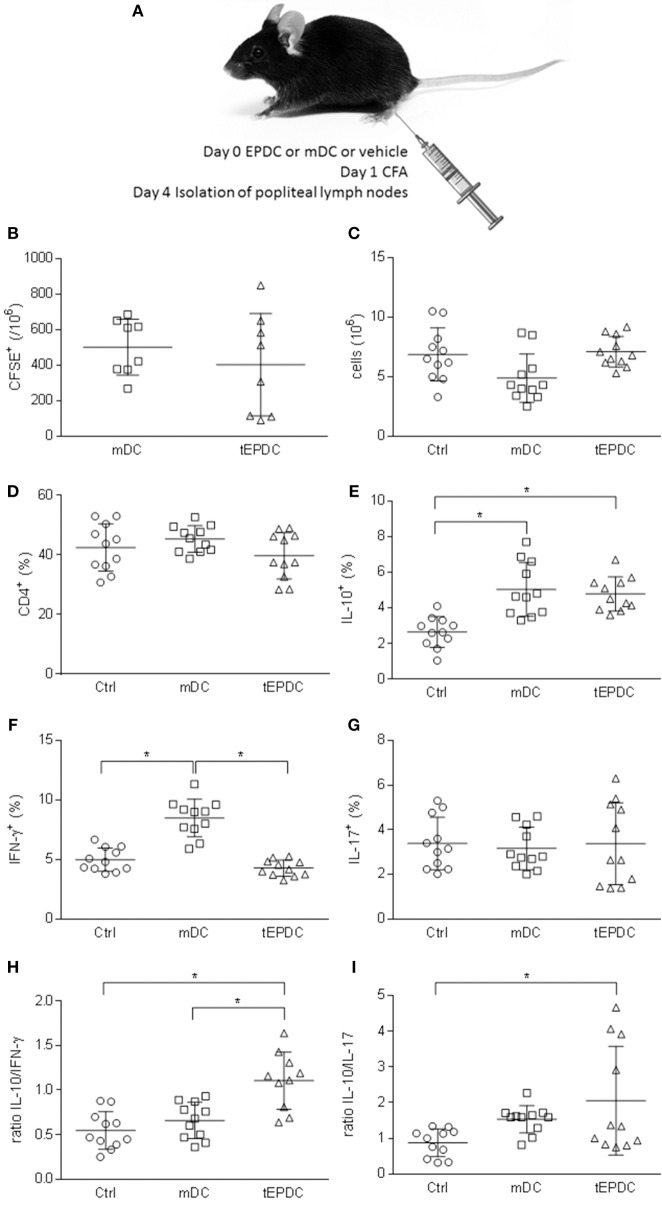
Effects of murine EP-treated DC on CFA-induced immune response *in vivo*. Schematic representation of C57BL/6 mice treatment **(A)**. Number of CFSE^+^ cells **(B)**, total number of cells **(C)**, and proportion of CD4^+^ cells **(D)** was determined in popliteal lymph nodes by flow cytometry **(B,D)** or cell counting **(C)**. Proportion of IL-10^+^ **(E)**, IFN-γ^+^ **(F)**, and IL-17^+^ **(G)** cells among CD4^+^ cells was determined by flow cytometry. Ratio of the cytokines expressing CD4^+^ cells is presented, as well **(H,I)**. Data from three experiments are presented as mean ± SD from a number of mice depicted as the circles, squares, or triangles. ^*^p < 0.05, one way ANOVA followed by Tukey's multiple comparisons test.

EPDC were applied *in vivo* to mice immunized with CFA as depicted in Figure 6A. Their effects were compared to those of mDC-treated mice, as well as to mice injected with vehicle only (control). In order to track DC *in vivo*, the cells were labeled with CFSE. CFSE-labeled EPDC and mDC were detectable in popliteal lymph nodes 4 days after the injection (Figure 6B). Popliteal lymph node cells were isolated for phenotypic analyses, at the same time. There were no differences in cellularity and proportion of CD4^+^ cells of popliteal lymph nodes isolated from vehicle-, mDC-, and EPDC-treated mice (Figures 6C,D). However, there were more IL-10^+^CD4^+^ cells in EPDC-treated than in control mice (Figure 6E) and less IFN-γ^+^CD4^+^ cells in EPDC-treated than in mDC-treated mice popliteal lymph nodes (Figure 6F). Both, IL-10^+^CD4^+^ cells and IFN-γ^+^CD4^+^ cells were also more numerous in mDC-treated than in control mice popliteal lymph nodes (Figures 6E,F). There were no differences in proportion of IL-17^+^CD4^+^ cells among the groups (Figure 6G). Importantly, ratio of IL-10^+^CD4^+^ cells to IFN-γ^+^CD4^+^ cells, as well as to IL-17^+^CD4^+^ cells was higher in popliteal lymph nodes of EPDC-treated mice in comparison to controls (Figures 6H,I). Moreover, ratio of IL-10^+^CD4^+^ cells to IFN-γ^+^CD4^+^ cells was higher in popliteal lymph nodes of EPDC-treated mice in comparison to mDC-treated mice.

## Discussion

Ethyl pyruvate modulates expression of antigen presentation-related molecules on DC. It also reduces release of pro-inflammatory cytokines from these cells. Moreover, it makes DC inefficient in T cell activation. Importantly, the effects are observed when EP is applied to DC during their differentiation/propagation from precursor cells, as well as when it is applied simultaneously with the maturation stimulus. EP-treated DC modulate immune response initiated by CFA *in vivo*. Thus, EP efficiently modulates immune activity of DC.

Our experiments on mice were performed with two mice strains, i.e., NOD and C57BL/6 mice. The reason to use both mice strains are our plans to expand the research to *in vivo* systems, where EAE and type 1 diabetes in mice will be analyzed. Both mouse strains are susceptible to EAE (12) induction, NOD mice develop diabetes spontaneously (13), while C57BL/6 mice are susceptible to multiple low dose streptozotocin-induced diabetes (14). Also, in this way we were able to exclude strain-specific effects of EP. The absence of genetic predisposition toward susceptibility of DC to EP were even more convincingly shown with human cells. There, the tolerizing effects of EP on DC were observed with the cells obtained from more than 10 different individuals. What is more, the effects were consistent with the cells obtained from healthy individuals, as well as with those obtained from individuals affected by multiple sclerosis. This implies that EP-treated DC are good candidates for a tolerogenic cell-based therapy. Along this conclusion, EP-treated DC were comparable to vitamin D3-treated DC, regarding their immunomodulating properties. The highest dose of EP applied in our study (12.5 mM) was even superior to standard vitamin D3 dose used (1 nM) when T cell proliferation was determined. Having in mind that vitamin D3-induced tolDC have been investigated in details *in vitro* and *in vivo* in EAE and that they are currently tested in clinical trials in multiple sclerosis, the ability of EP to act on DC in similar fashion is an additional impetus for further studies toward clinical application of EP-treated DC as a therapy for autoimmunity.

Interestingly, there was a difference in IL-10 production in the co-cultures of tolDC with PBMC, depending on the source of DC, i.e., whether the cells were obtained from healthy or multiple sclerosis individuals. While IL-10 levels were increased in the co-cultures of tolDC from healthy individuals, they were not increased in the co-cultures of tolDC from multiple sclerosis individuals. This discrepancy was observed irrespectively of the tolerizing agent used (EP or vitamin D3). It is proposed that IL-10 plays the crucial role in immunomodulatory activity of tolDC, as it induces differentiation of regulatory T cells (15). Thus, the observed phenomenon is worthy of further investigation, as it could be potentially disadvantageous to therapeutic potential of EP- and vitamin D3-induced tolDC. Also, the possibility of inherent reduced capacity of DC and other immune cells of multiple sclerosis patients to produce IL-10 warrants additional studies as it might be important for understanding the predisposition of humans toward the development of multiple sclerosis. Indeed, reduction in the proportion of IL-10-producing PBMC in multiple sclerosis patients (16), as well as of IL-10 mRNA expression in these cells (17, 18) were previously reported. However, multiple sclerosis patients and healthy subjects from whom blood samples were obtained were not adjusted to age, sex, and other relevant parameters that could bias our observation on the differential IL-10 response. Also, higher number of samples would be needed for the determination of the statistical correlation between the levels of cell proliferation and IL-10 or IFN-γ production within groups of healthy subjects and multiple sclerosis patients, as well as between these two groups. Therefore, further studies enrolling higher number of individual samples for the exploration of the effects of EP on human DC, including their ability to generate IL-10 are needed. However, the fact that the samples were not adjusted between multiple sclerosis and healthy subjects did not jeopardize the general conclusion about tolerizing potency of EP-treated DC, i.e., EP-treated DC had the same, if not stronger tolerizing effects in comparison to classical tolDC induced by vitamin D3, irrespectively if they were obtained from the healthy subjects or the patients.

EP-treated DC applied to mice immunized with CFA shifted draining lymph node cytokine milieu from IFN-γ/IL-17- toward IL-10-dominated. EP-treated DC injected at the site of injection efficiently migrated toward regional lymph nodes and imposed the immunomodulatory effect. The observed modulation of the cytokine-generating T cell balance is particularly fascinating, as it was observed in strong inflammatory setting induced by CFA. Thus, immunomodulatory effects of EP-treated DC are rather potent and the one can expect that the effects would persist in antigen-specific setting, as well. Of course, in the context of a certain autoimmune disease it would be important to determine if EP-treated DC loaded with relevant antigens would acquire specificity of immunomodulatory actions. Thus, our results imply that EP-treated DC dampen initiation and propagation of an immune response in lymphoid organs, an effect that should be useful in restricting the immunopathology of autoimmune and chronic inflammatory diseases. This possibility will be explored in details in specific models of autoimmunity, such as type 1 diabetes in NOD mice and EAE in our future studies.

We have previously shown that EP exerts immunomodulatory properties in animal models of multiple sclerosis and type 1 diabetes (6, 19). Here we extend our observation and we introduce a novel biological effect of EP, i.e., its ability to induce tolerogenic DC *in vitro*. Moreover, EP-treated DC are efficient in modulating an immune response *in vivo*. EP applied to DC during the process of differentiation inhibits their response to the maturation stimulus, both regarding expression of antigen-presenting and co-stimulatory molecules and production of relevant pro-inflammatory cytokines. Also, EP-treated DC are impotent allogeneic stimulators of CD4^+^ T cells. Importantly, the effects are observed in human cells and not only murine cells. Further studies are needed for determination of molecular mechanisms behind the effects of EP. Also, *in vivo* studies on animal models of autoimmunity are necessary. These studies should help to elucidate if EP-treated DC are good candidates for clinical trials of multiple sclerosis and other autoimmune disorders.

## Author Contributions

ND performed the main work, analyzed, interpreted, and critically revised the data. MM performed the work related to MDDC. BJ organ isolation, cell preparation, cytofluorimetry. JN-B performed the work related to MDDC. TS organ isolation, co-culture, CD4^+^ T cell purification. EM-C conception and design, supervised the work, and critically revised the manuscript. ÐM conception and design, supervised the work, analyzed and interpreted the data, drafted the work, and critically revised the manuscript. All authors finally approved the version to be published and agreed to be accountable for all aspects of the work in ensuring that questions related to the accuracy or integrity of any part of the work are appropriately investigated and resolved.

### Conflict of Interest Statement

The authors declare that the research was conducted in the absence of any commercial or financial relationships that could be construed as a potential conflict of interest.

## Abbreviations

7AAD: 7-Amino-actinomycin D
BMDC: bone marrow derived dendritic
CD: cluster of differentiation
CFA: complete Freund's adjuvant
CNS: central nervous system
DC: dendritic cells
diffEP: DC trated with EP during differentiation/propagation
EAE: experimental autoimmune encephalomyelitis
EP: ethyl pyruvate
FCS: fetal calf serum
GM-CSF: granulocyte-macrophage colony-stimulating factor
HLA: human leukocyte antigen
iDC: immature DC
IFN: interferon
IL: interleukin
LPS: lipopolysaccharide
matEP: DC treated with EP during maturation
mDC: mature DC
PBMC: peripheral blood mononuclear cells
PBS: phosphate buffer saline
SD: standard deviation
Th: helper T cells
TNF: tumor necrosis factor
tolDC: tolerogenic DC
Treg: regulatory T cells.
